# High Allelic Heterogeneity in Kazakhstani Patients with Neurofibromatosis Type 1: Results from the First Molecular Study

**DOI:** 10.3390/genes16111390

**Published:** 2025-11-19

**Authors:** Zhannat Idrissova, Farida Rakhimbekova, Madina Orazgaliyeva, Madina Zhaksybek, Kristina Kovaleva, Saltanat Abdikerim, Aizhan Ormankyzy, Petr Vasiluev

**Affiliations:** 1 Competence Center for Neurological Orphan Diseases, University Clinic Aksai, Asfendiyarov Kazakh National Medial University, Almaty 050067, Kazakhstan; 2 Genomic Laboratory, Asfendiyarov Kazakh National Medial University, Almaty 050012, Kazakhstan; farida.rakhimbekova@yandex.com (F.R.); kovaleva.chr@gmail.com (K.K.); 3 Center for Molecular Genetic Research, Kazakh Institute of Oncology and Radiology, Almaty 050022, Kazakhstan; madina259@mail.ru; 4 Department of Neurology, University Clinic Aksai, Asfendiyarov Kazakh National Medial University, Almaty 050067, Kazakhstan; zhaksybek_madina@mail.ru; 5 Institute of Genetics and Physiology, Almaty 050060, Kazakhstan; abdikerimse@gmail.com; 6 Department of Medicine, Astana Medical University, Almaty 010000, Kazakhstan; aizhan.ormankyzy8@gmail.com; 7 Academician N.P. Bochkov Research Centre for Medical Genetics (RCMG), Moscow 115522, Russia; fbmmsu33@gmail.com

**Keywords:** neurofibromatosis 1, Kazakh cohort, plexiform neurofibroma, nonsense variants, missense variants

## Abstract

**Background/Objectives:** This study presents the first molecular characterization of *NF1* gene variants in Kazakhstani patients, expanding regional understanding of neurofibromatosis type 1 (NF1). The *NF1* gene encodes *neurofibromin*, a tumor suppressor protein that regulates the MAPK signaling pathway; its inactivation results in NF1, a multisystem disorder with pigmentary and tumor manifestations. **Methods:** A total of 60 pediatric and young adult patients of University Clinic Aksai were selected based on Legius criteria and studied clinically; genetic variants of *NF1* gene were determined with AmpliSeq for Illumina Myeloid Panel (next generation sequencing). **Results:** Pathogenic or likely pathogenic (with some variants of unknown significance) were detected in 58 of 60 (96.7%) patients. Among them, 27 (46.6%) carried point variants, 21 (36.2%) had genomic deletions, 3 (5.2%) had duplications, 3 (5.2%) insertions, and 4 (6.9%) had exon–intron splicing site variants. Notably, all patients with duplication insertions and splicing variants presented with plexiform neurofibromas. **Conclusions:** The study defines the first variant spectrum in a Kazakhstani population, confirming genotype–phenotype correlations consistent with European cohorts (l.). These data highlight the predominance of structural and splicing alterations in patients with plexiform neurofibromas and support the integration of molecular testing into clinical management of NF1 in Kazakhstan.

## 1. Introduction

*NF1* encodes the large tumor suppressor protein *neurofibromin,* which regulates the MAP-kinase signaling pathway [1]. *Neurofibromin* acts as *Ras GTPase-activating* protein (GAP) that inactivates *Ras-GTP* (*guanosine triphosphate), Ras GTP,* thereby controlling cell growth [2]. Pathogenic variants in *NF1* disrupt this regulation, leading to persistent Ras activation and excessive signaling through the MAP cascade, which promotes uncontrolled cell proliferation and oncogenic transformation [3].

Pathogenic variants in the *NF1* gene (autosomal-dominant with full penetration) cause neurofibromatosis 1 type (NF1) [4]. It is characterized by café-au-lait macules across the body, neurofibromas and plexiform neurofibromas, optic gliomas, Lisch nodules, seizures, intellectual disabilities and learning difficulties, scoliosis and osteoporosis as well as other multi-organ manifestations [5,6]. Neurofibromas are benign tumors that grow along the neural sheaths across the body, causing pressure and displacement of adjacent tissues and organs [7]. Plexiform neurofibromas, found in 40–50% of all neurofibromatosis 1 patients, are thick and misshaped and can occur at any part of the body [7]. Patients describe neurofibromas as painful and uncomfortable, leading to surgical removal under surgeons and treatment team agreement. In approximately 15–30% cases, plexiform neurofibromas transform to malignant peripheral nerve sheath tumors (MPNSTs) [6,7].

Common variants that lead to *NF1* gene dysfunction are single nucleotide polymorphism (SNP), including single amino acid replacements and premature stop codons, insertions, deletions, splicing area variants, and indels [3]. Modern diagnostics of neurofibromatosis, along with patient history and visualization techniques, include the sequencing of the *NF1* gene [8].

The standard of care in neurofibromatosis 1 includes genetic testing, which the differentiation of this condition from other phacomatoses and admits patients with pronounced plexiform neurofibromas into target therapy [5]. Search for variants might include methods of next-generation sequencing, less often MLPA (multiplex ligation-dependent probe amplification), and more rarely Sanger sequencing and karyotyping [8].

Next-generation sequencing (NGS) is a common method of detection of genomic variants in patients who have a confirmed status of café au lait macules, neurofibromas, vision, and skeleton manifestations [9,10]. In this study, NGS was employed to comprehensively analyze all *NF1* exons and canonical splice sites, enabling the detection and characterizations of a broad range of genomic alterations in patient with neurofibromatosis 1 [11].

This work is the first study in Kazakhstan that considers neurofibromatosis. To complete this investigation, we received a research grant from the Ministry of higher education of Republic of Kazakhstan, who are particularly interested in the research of rare genetic diseases in the country. This study adds to the understanding of neurofibromatosis in an under-represented population. Furthermore, this study aimed to establish a cycle of *NF1* patient care from diagnosis to treatment via clinical, instrumental, and NGS methods.

## 2. Materials and Methods

Pediatric patients admitted to Aksai University Clinic, division of Anfendiyarov Kazakh National Medical University (Asfendiyarov University) with diagnosis neurofibromatosis 1 or complaints matching diagnosis criteria of NF 1 were selected for sequencing. The following inclusion diagnostic criteria (Legius criteria) were used [12]:

- Six or more café-au-lait macules on the body, sized ≥ 5 mm;
- Two or more neurofibromas or one plexiform neurofibroma visualized on MRI (magnetic resonanse imagin;
- Bilateral multiple lentigos (freckles) localized in large skin folds (axillary and/or inguinal);
- Two or more Lisch nodules on the iris, two or more choroidal abnormalities;
- Optic pathway glioma.

Children were admitted to both hospital inpatient and outpatient during the years of 2023–2025. A total of 60 patients of ages 1.5 to 25 (children and young adults) were studied.

The study was conducted in accordance with the Declaration of Helsinki, and the protocol was approved by the Local Ethics Committee of Asfendiyarov University, AP19676226 on 4 November 2022, LEC № 11 (134).

Venous blood was collected in EDTA K2 tubes. DNA extraction was performed at the Genomic Laboratory, Asfendiyarov University. Samples were maintained at +4 °C under cold-chain conditions. A KingFisher Flex system with Ready DNA Ultra 2.0 Prefilled Plates (Thermo Scientific, Waltham, MA, USA) was used to extract genomic DNA from blood (200 µL per sample). DNA concentrations and quality were checked on NanoDrop One/One C, Qubit (Thermo Scientific, USA). DNA was stored at −20 °C until further experimental application. A total of 10 ng of DNA per sample was used for library preparation with the AmpliSeq for Illumina Myeloid Panel (Illumina, San Diego, CA, USA). Library size distribution and quality were verified using the High Sensitivity DNA Chips on the Agilent 2100 Bioanalyzer System, 2100 Expert Software Revision B.02.12 (SR2). Copy number variations (CNV) were not examined in this study.

Statistics: sequencing data was analyzed with BaseSpace Annotation Engine and VariantStudio from Illumina. Spice-donor area variants were included in the analysis; other deep (more than 10 nucleotides) variants were excluded due to lack of the description and relevance in the literature. Data was sorted according to variant profile and clinical representation of patients. Loss of function and missense variants were compared according to the severity of manifestation using Fisher’s exact test.

Missense variants were further analyzed using MutationMapper from cBioPortal, v6.4.1 (https://www.cbioportal.org/mutation_mapper, accessed on 15 October 2025).

## 3. Results

Out of 60 probands from non-related families, 58 individuals had the altered genomic variant in *NF1* gene; most of the found variants had been described in databases before (NCBI, Varsome, Lausanne, Switzerland), though some were novel (Supplement Tables S1–S3). The general characteristics of the investigation cohort were as follows: among patients, there were children and adolescents of ages ranging from 1.5 to 25 years (over 18 years—two patients); the majority were children who were aged 4 to 16 years old (54 cases), with two children aged 1.5 years. Of the 58 children with confirmed *NF1* gene diagnosis, 27 of them were boys and 31 were girls; accordingly, the ratio was 47% males to 53% females. The observed patients had no history of alcohol consumption and smoking. Body mass index (BMI), was normalized according to age. A normal BMI range for children 1.5–10 years old was considered as 14–18; in the two adolescences that were 15- and 17-years-old, their BMIs were 25, which was higher than 1 standard deviation (Sd), but was less than 2 Sd. The glucose level in the 56 patients examined was within the normal range and did not exceed 6 mmol/L; one girl had a slight increase in their glucose level to 6.7 mmol/L, while in one child with an established diagnosis of diabetes mellitus who used an insulin pump, their glucose level reached 6.7–7.6 mmol/L. Alanine aminotransferase (ALT) levels were normal during all clinical examinations (up to 40 U/L), while aspartate aminotransferase (AST) was slightly elevated (up to 50 U/L) in four children with biliary dyskinesia. C-reactive protein levels were transiently 1.5 times higher than normal (up to 7.5 mg/mL) in four children with respiratory infection symptoms; these levels then normalized to 5 mg/mL, and this level was subsequently maintained. ESR (erythrocyte sedimentation rate) was within normal limits: in 55 children, it did not exceed 15 mm/h, in 3 children, it was 18–19 mm/h, while in the case of 1 17-year-old girl, their ESR was 20 mm/h; she also noted pain in the lumbosacral region, where a plexiform neurofibroma was localized.

The study shows that 21 of the children have deletion genomic variants, and 27 have point variants (out of 58). There were a further three variants of duplication, three of insertion, and four in the splicing site. Variants were classified as either nonsense and missense and the separate group comprised the splicing variants. Among patients with duplications, in three cases of three children, (100%) there were diagnoses with plexiform neurofibromas; of three patients with insertion variants, three had plexiform neurofibromas (100%); and among four children with exon–intron splicing site variants, all four (100%) had plexiform neurofibromas.

Patients exhibited clear manifestations of neurofibromatosis with café-au-lait stains in all cases. The majority of cases had simple neurofibromas (34 cases, 59%) and plexiform neurofibromas (PlNf) (41 cases, 71%), as well as axillary freckling. The other manifestations were not so common; additionally, optic nerve involvement (thickening, glioma, and retinopathy) was observed in a quarter of cases, with the former appearing less (Table 1).

**Figure 1:**
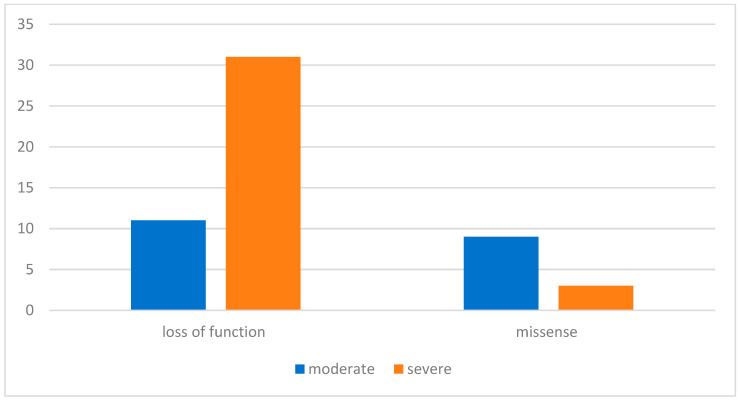
Distribution of NF1 gene variants in pediatric patients of Aksai cohort.

The variants identified were predominantly loss of function (LoF) (including deletions, duplications, insertions, and variants at exon–intron splicing zone)—79%—followed by missense—21%. As shown in Figure 1, in the LoF cohort, 76% of patients had severe manifestations of neurofibromatosis (pronounced plexiform neurofibromas, high grade 3 or 4 scoliosis with definite motor symptoms, back pain, gliomas, and other corresponding symptoms, such as epilepsy); 24% had a lighter course of the disease (mostly café-au-lait macules). In the missense cohort, 25% had severe manifestations and 75% had moderate manifestations. In the comparison of nonsense patients versus missense *NF1* variants, the use of Fisher’s exact test demonstrated that the relative risk (RR) of developing a severe clinical phenotype in patients with nonsense variants was 8.45 times higher than those with the missense variants (*p* = 0.003). Comparable results were reported by other authors [13].

**Figure 2:**
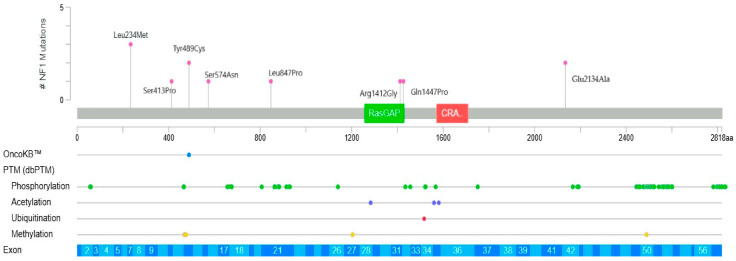
Localization of single nucleotide variants (missense) on *NF1* gene (12 patients). The Ras-GAP domain is shown in green, C-terminal CRAL-TRIO (CRA) domain is shown in red.

Patients carrying missense variants are shown in Figure 2, where each variant is positioned along the *NF1* gene from 5′ to 3′ end. A single nucleotide variant, c.700C>A, located near the 5′ region of the *NF1* gene, may have caused reduced or absent synthesis of neurofibromin. Clinically, this variant was detected in one patient with large plexiform neurofibroma of the pelvis, one with a moderate PlNf of the pancreas, and one with a small PlNf of the chest. Two additional variants studied near the Ras-GAP binding domain were observed in patients with café-au-lait macules and symptomatic epilepsy. The Tyr489Cys variant was identified in two patients with café-au-lait macules and in one patient with small PlNf; this variant is annotated in public databases as likely a loss-of-function. Four other variants located near phosphorylation-regulatory regions were associated with milder clinical manifestations in our cohort.

The observed café au lait macules originated in the early childhood of our patients and in most cases grew as they approached adolescence. Around 60% of patients with neurofibromatosis had two, simpler neurofibromas, characterized by nerve hypertrophy, which in turn caused displacement of adjacent tissues, organs, and bones if located next to the pelvis and/or spinal cord. There were several patients presenting with severe cases of scoliosis, lordosis, and torsion of the rib cage due to ongoing growth of neurofibromas, identified via MRI as focal areas of increased signal intensity, with space-occupying lesions verified on the T2-MRI STIR (Short Tau Inversion Recovery) regime as plexiform neurofibromas.

**Figure 3:**
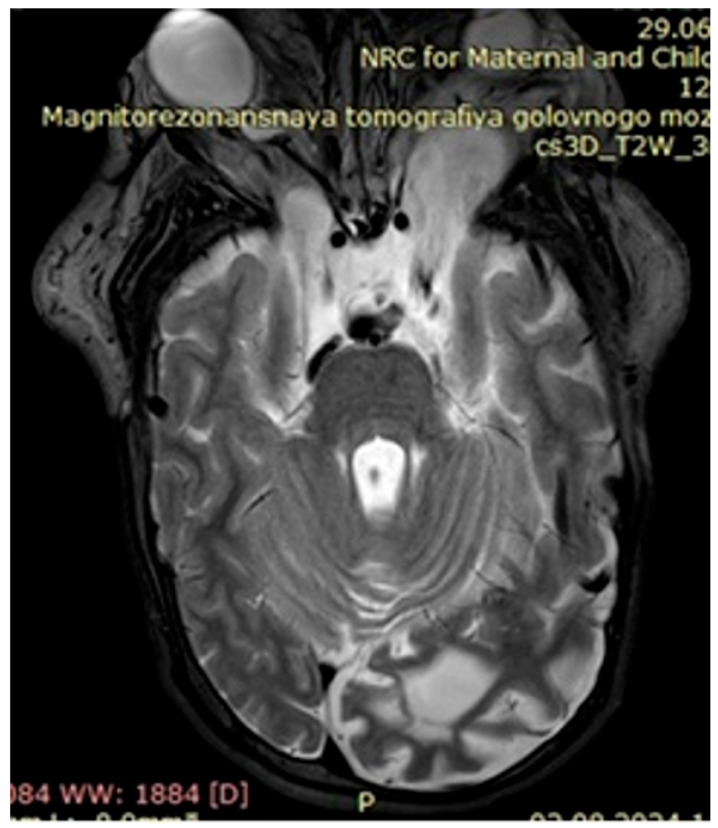
Case 1, clinical diagnosis of a 14-year-old girl: neurofibromatosis 1, full facial plexiform neurofibroma, especially eye lids, forehead, and the orbits of both eyes, with compression of the eyeballs. With the deletion of the *NF1* gene Chr17, deletions NM_000267.4:c.3843delC.

**Figure 4:**
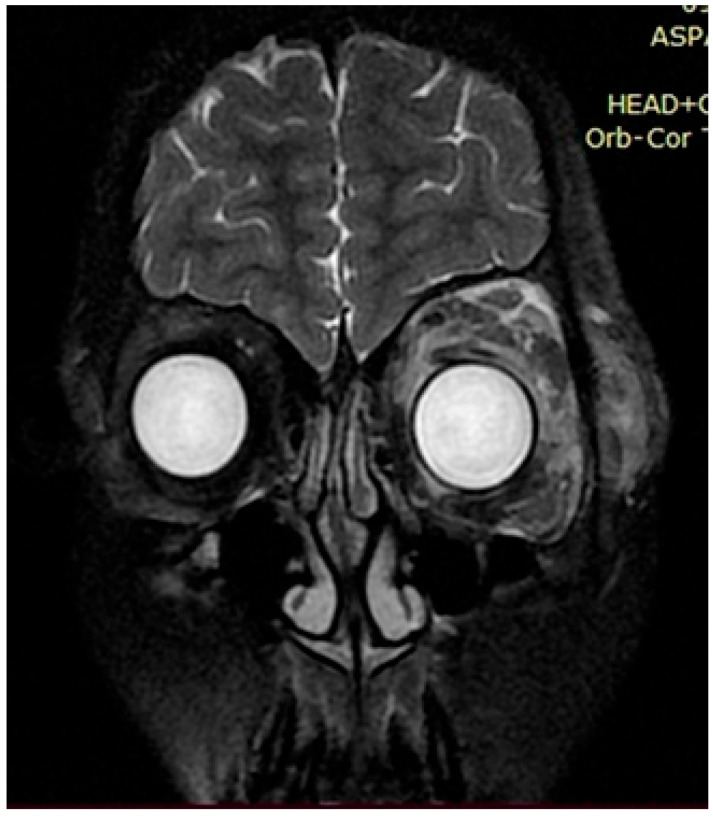
Case 2, clinical diagnosis of a 12-year-old boy: neurofibromatosis type 1, plexiform neurofibroma of the left periorbital region, mild truncal ataxia, and mild dysmetria. With single nucleotide replacement variant *NF1* Chr17 NM_000267.4:c.5792G>A.

**Figure 5:**
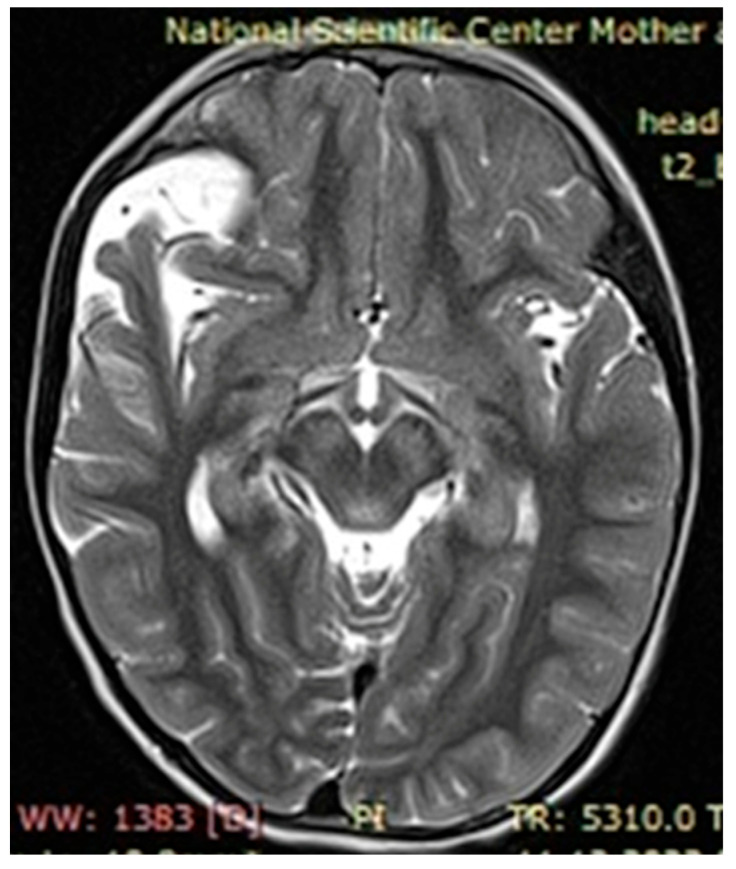
Case 3, clinical diagnosis of a 6-year-old girl 6: neurofibromatosis type 1, plexiform neurofibroma at the level of the right greater wing of the sphenoid bone with dysplasia, narrowing of the right retrobulbar space, with compression of the right optic nerve (over a length of 1.0 cm) and the superior and lateral rectus muscles of the right eye. *NF1* gene genotyping: Chr17: 29576060 NM_001042492.2:c.4033A>T p.Lys1345Ter.

Upon further growth of neural tissues, patients presented with plexiform neurofibromas, located around the eye tract, neck, spinal cord, and groin (examples presented in Figure 3, Figure 4 and Figure 5). A total of 14 patients presented with optic nerve and optic tract glioma as well as retinopathies, retinal angiopathies, declined vision, astigmatism, and other eye pathologies. Many patients complained that eye plexiform neurofibroma occluded their vision, causing gradual vision loss due to lack of accommodation and increased tissue growth.

Seizures and abnormal EEG (electroencephalography) activity was presented in nine patients with focal seizures and in some patients with tonic–clonic seizures. Liver and pancreas disorders, including changes in liver parenchyma, biliary dyskinesia, and reactive pancreatitis, were present in 33% of patients.

## 4. Discussion

Deletions, duplications, and insertions caused frameshift variants leading to the formation of a premature stop codon downstream in the gene. In the *NF1* gene, this results in nonsense-mediated mRNA decay and the absence of a functional *neurofibromin*. A lack of *neurofibromin* keeps the Ras protein active all the time, leading to proliferation of tissues.

*Neurofibromin* acts as GTPase-activating protein (GAP) for Ras; therefore, its loss prevents Ras inactivation and maintains Ras in the GTP-bound active state, driving persistent proliferative signaling.

The absence of *neurofibromin* leads to persistent Ras activation and continuous stimulation of MAPK and PI3K/AKT signaling. This dysregulated growth control promotes the formation of plexiform neurofibromas.

Patients with nonsense variants had more plexiform neurofibromas, as confirmed by Fisher’s exact test; in many cases, these were of a large size and in locations that pose a threat to vital organs (such as the eyes, mediastinal structures, or the spinal cord, with a risk of fatal compression).

Ponti et al. (2014) describes the p.(Arg440*) variant as having elephantiasis neuromatosa, neurofibromas, and café au lait macules [14]. This compares to the p.(Arg440*) variant in our cohort with gigantic plexiform neurofibromas and malignant transformation. Ponti et al. further describe c.1541_1542del: this is found in elephantiasis neuromatosa, neurofibromas, hamartomas, scoliosis, and café au lait macules. Our four patients with NF1 also had café au lait macules, plexiform neurofibromas, gliomas, and scoliosis. In Ponti et al., p.(Leu1153Metfs*4) is associated with elephantiasis neuromatosa, neurofibromas, hearing loss, and café au lait macules. In our cohort, this variant is associated with café au lait macules, simple neurofibromas, angiopathy, and pancreatitis. Overall, variants described in Ponti et al. and in our study have matching clinical manifestations which could indicate a genotype–phenotype correlation.

Melloni et al. (2019) describes p.Ser1282Valfs3* with optic pathway glioma; in our study, the patient with the p.Ser1282Valfs3* variant had paraorbital plexiform neurofibroma [15]. p.(Arg440*) is described in Melloni et al. also, being found in optic pathway glioma. p.Tyr489Cys in Melloni et al. and in our study is associated with optic pathway glioma.

The missense variant *NF1* p.(Tyr489Cys) was observed in our cohort. This variant is well-documented in multiple published cohorts (including Melloni et al.) and is classified as pathogenic in public databases including ClinVar (VCV000000354). RNA-based studies demonstrate that the c.1466A>G substitution activates a cryptic splice donor site, resulting in the skipping of 62 nucleotides of exon 13 and a truncated *neurofibromin* product. Given the recurrent observation in NF1-affected individuals and the evidence of disrupted splicing, the variant is considered disease-causing [16].

Elefanti et al. (2021) describes p.Lys1345Ter, found in nodular melanoma and acral lentiginous melanoma; in our study, this variant is associated with plexiform neurofibroma, optic nerve glioma, and femoral shortening [17]. Other loss of function variants were in some cases presented in genomic databases, but without peer-reviewed case reports.

In our cohort, we identified a single-nucleotide variant, c.700C>A, located near the 5′ region of the *NF1* gene. This substitution is predicted to impair or abolish *neurofibromin* synthesis, consistent with the severe clinical manifestations observed in the three unrelated patients, who presented with large, moderate, and small plexiform neurofibromas of the pelvis, pancreas, and chest, respectively. To the best of our knowledge, this variant has not been previously reported in public databases such as ClinVar, LOVD, or HGMD, suggesting it may represent a novel pathogenic alteration. According to the ACMG guidelines, this variant meets the criteria PM2 (absent from population databases), PP3 (multiple lines of computational evidence support a deleterious effect), and PP4 (phenotype highly specific for NF1-related disease), and can therefore be classified as likely pathogenic. Further studies are required to confirm its pathogenic role.

The missense variant *NF1* c.1237T>C (p.Ser413Pro) was identified in our cohort. To our knowledge, this variant has no dedicated peer-reviewed case report, but it appears in public databases (ClinVar Alllele Regisrty ID CA398997666, rs1555611093) and in our study, where it was associated with plexiform neurofibroma.

The *NF1* c.1721G>A (p.Ser574Asn) variant affects the last nucleotide of exon 15, a position critical for splice donor recognition. Stella et al. (2018) [18] demonstrated that this substitution disrupts the canonical donor site, leading to aberrant mRNA splicing and likely the nonsense-mediated decay of the transcript. Consistent with these molecular data, Kirat and Mutlu Albayrak (2021) [19] classified this variant as pathogenic based on multiple ACMG criteria (PM1, PM2, PM5, PP2, PP3, and PP5) in a Turkish NF1 cohort. In our patient, this variant was associated with a plexiform neurofibroma located extracranially above the processus mastoideus, supporting its functional relevance in *neurofibromin* loss.

The *NF1* c.2540T>C (p.Leu847Pro) variant has been previously reported in several cohorts of patients with neurofibromatosis 1 type. Tabata et al. (2020) [20] described the missense change in two unrelated individuals presenting with plexiform neurofibromas and developmental delay, further supporting its pathogenic significance. Pacot et al. (2025) [21] further describes this variant as associated with Noonan-like features, including developmental delay. This substitution affects a conserved leucine residue within a region essential for structural stability and Ras-GTPase regulatory function. In our patient, this variant was associated with optic nerve glioma, strabismus, and developmental delay.

The *NF1* c.4234A>G (p.Arg1412Gly) variant affects a conserved residue within the GAP-related domain of *neurofibromin*, a known mutational hotspot where multiple substitutions at Arg1412 have been reported as pathogenic. This amino acid plays a key role in Ras-GTPase regulation, and its alteration is likened to the loss of *neurofibromin* function. In our patient, the variant was associated with symptomatic epilepsy, intellectual disability, and simple neurofibromas, further supporting its clinical significance in NF1-related neurological manifestations.

Th *NF1* c.4340A>C (p.Gln1447Pro) variant affects a conserved residue within the GAP-related domain of *neurofibromin*, at a codon previously recognized as pathogenic, where other substitutions such as p.Gln1447Arg, p.Gln1447His, and p.Gln1447Ter have been described in patients with neurofibromatosis 1. The recurrence of pathogenic alterations at this position indicates its structural and functional importance in Ras-GTPase regulation, supporting the clinical relevance of the p.Gln1447Pro change observed in our study.

The *NF1* c.6401 A>C (p.Glu2134Ala) variant has been cataloged in the ClinGene Allele Registry as affecting a conserved residue in the C-terminal region of *neurofibromin*. Although there is no case report publication, its location downstream of the Gap-related domain suggests it may disrupt secondary regulatory or structural functions of *neurofibromin*.

Safonov et al. (2024) [22] employed a genotype-first approach using a large biobank cohort and identified a high incidence of pathogenic *NF1* variants with diverse systemic associations. In contrast, our study was based on a clinic-derived cohort from the Aksai clinic, where patients were primarily referred for neurological and orthopedic manifestations. Consequently, our findings reflect the phenotypic spectrum typical of a specialized clinical population, rather than the general Kazakh population. Despite this difference in sampling design, both studies underscore the wide clinical variability of NF1 and highlight the importance of expanding molecular diagnostics across different populations and healthcare settings.

In our study of the Kazakh NF1 cohort, no breast cancer cases were identified—a finding consistent with the possibility that our sample (composed primarily of children and young adolescents, and selected for neurological/orthopedic presentations) may not yet capture the adult-onset tumor spectrum highlighted by Frayling et al. [23].

In conclusion, this study provides the first molecular characterization of NF1 in Kazakh patients, revealing a diverse spectrum of pathogenic variants and highlighting the need for broader population-based research to fully define genotype–phenotype correlations and improve genetic diagnostics across Central Asia.

## Figures and Tables

**Table 1:** Stratification of patients according to the observed Legius criteria and other NF1 manifestations (out of 58 patients).

Legius Criteria and Other NF1 Manifestations	Number of Patients	% From Total
Six or more café-au-lait stains	58	100%
Two or more cutaneous/subcutaneous neurofibromas	34	59%
Plexiform neurofibromas	41	71%
Axillary freckling	45	78%
Bone distortions (scoliosis, lordosis, and torsion of the rib cage)	11	19%
Optic nerve thickening/glioma/retinopathy	14	24%
Strabismus	3	5%
Lisch nodules	2	3%
Neurodevelopmental delay	20	34%
Liver and pancreas disorders	19	33%
Seizures	9	16%
Cardiomyopathy	4	7%
Paresis	2	3%

## Data Availability

The original contributions presented in this study are included in the article/Supplementary Materials. Further inquiries can be directed to the corresponding author.

## Supplementary Materials

The following supporting information can be downloaded at https://www.mdpi.com/article/10.3390/genes16111390/s1: List of genes of Myeloid Panel, Table S1: Nonsense variants identified in NF1 patient cohort, Table S2: Single nucleotide substitutions (missense variants) identified in NF1 patient cohort, Table S3: Exon–intron splicing site variants in patients with NF1.

## Abbreviations

The following abbreviations are used in this manuscript:

NF	Neurofibromatosis
EEG	Electroencephalogram
ADHD	Attention deficit hyperactivity disorder
PlNf	Plexiform neurofibromas
MRI	Magnetic resonance imaging
BMI	Body mass index
ESR	Erythrocyte sedimentation rate
ALT	Alanine aminotransferase ALT
AST	Aspartate aminotransferase AST
