# Supplementary material for: High Allelic Heterogeneity in Kazakhstani Patients with Neurofibromatosis Type 1: Results from the First Molecular Study
Source: Genes (Basel). 2025 Nov 19;16(11):1390. doi: 10.3390/genes16111390 (PMC12652172; doi:10.3390/genes16111390)
Supplement: Supplementary file 1 [file genes-16-01390-s001.zip › genes-3983285-supplementary.pdf]

Supplement Materials

List of genes of Myeloid panel: *ASXL1, BCOR, CALR, CEBPA, ETV6, EZH2, IKZF1, NF1, PHF6, PRPF8, RB1, RUNX1, SH2B3, STAG2, TET2, TP53, ZRSR2*.

Abbreviations: cafe au lait macules (c-l m); optic nerve (ON); Plexiform neurofibromas (PlNf); simple neurofibromas (SNF); Focal areas of signal intensity (fasi) in brain MRI; ADHD – attention deficit hyperactivity disorder; EEG – electroencephalogram.

Table S1. Nonsense variants identified in NF1 patient cohort.

Genomic	Variants	Protein	Repeats	Pathogenicity	Clinical features
NC_000017.11:<br>g.31223478_31223481del	NM_001042492.3: c.1756_1759del	NP_001035957.1:p.(Thr586ValfsTer18)	3	**Pathogenic**<br>PVS1, PM2, PS4, PP5	c-l m<br>SNf<br>ADHD<br>PlNf<br>(3 patient)
NC_000017.11:<br>g.31349185_31349186del	NM_001042492.3: c.7255_7256del	NP_000258.1: p.(Leu2398GlyfsTer2)	2	**Pathogenic**<br>PVS1, PM2, PS4, PP5	PlNf<br>(2 patients)<br>SNf<br>& Scoliosis<br>& Paraparesis<br>c-l m
NC_000017.10:g.29546036_29546037del	NM_001042492.3: c.1541_1542del	NP_001035957.1:p.(Gln514ArgfsTer43)	4	**Pathogenic**<br>PVS1, PS2, PM2, PP5	c-l m<br>PlNf<br>(4 patients)<br>angiopathy<br>ON glioma

					Scoliosis
NC_000017.11:g.31260427_31260431del	NM_001042492.3:c.4489_4493del	NP_001035957.1:p.(Ser1497HisfsTer3)	1	**Pathogenic**<br><br>(PVS1, PM2, PP5)	PlNf<br><br>(c-l m<br><br>Lisch nodules<br><br>optic nerve (ON) glioma<br><br>strabismus
NC_000017.10:g.29486069_29486070del	NM_001042492.3:c.246_247del	NP_001035957.1:p.(Gln83ValfsTer23)	2	**Pathogenic**<br><br>PVS1, PM2, PS4, PP5	PlNf (2 patients)<br><br>c-l m. &<br><br>paraparesis<br><br>hyperkinesis
NC_000017.11:g.31232716del	NM_001042492.3:c.3331del	NP_001035957.1:p.(Met1111Ter)	1	**Pathogenic**<br><br>PVS1, PM2, PP5	Paraparesis<br><br>SNf<br><br>PlNf<br><br>scoliosis<br><br>spike-slow wave complex (EEG)
NC_000017.11:g.31229880del	NM_001042492.3:c.2896del	NP_001035957.1:p.(Ala966LeufsTer2)	1	Likely Pathogenic<br><br>PVS1, PM2<br><br>(novel variant)	c-l m<br><br>Mental retardation<br><br>Cardiomyopathy<br><br>OM glioma

NC_000017.11:g.31235745del	NM_001042492.3:c.3843del	NP_001035957.1:p.(Ser1282ValfsTer3)	1	Likely Pathogenic<br><br>PVS1, PM2<br><br>novel variant	PlNf paraorbital
NC_000017.11:g.31232842_31232845del	NM_001042492.3:c.3457_3460del	NP_001035957.1:p.(Leu1153MetfsTer4)	1	**Pathogenic**<br><br>PVS1, PM2, PS4, PP5	c-l m<br><br>SNf<br><br>Pancreatitis
NC_000017.11:g.31327782_31327786del	NM_001042492.3:c.5552_5556del	NP_001035957.1:p.(Pro1851HisfsTer9)	1	Likely Pathogenic<br><br>PVS1, PM2<br><br>(novel variant)	c-l m<br><br>Mental retardation<br><br>Fasi
NC_000017.11:g.31334932_31334935del	NM_001042492.3:c.5907_5910del	NP_001035957.1:p.(Arg1970LeufsTer8)	1	Pathogenic<br><br>PVS1, PM2, PS4, PP5	c-l m<br><br>Fasi<br><br>Symptomativ epilepsy<br><br>ON glioma
NC_000017.11:g.31229030_31229040del	NM_000267.3:c.2415_2425del	NP_000258.1:p.(Glu806GlnfsTer5)	1	Likely Pathogenic<br><br>PVS1, PM2<br><br>(novel variant)	c-l m &<br><br>simple Nf<br><br>scleroderma
NC_000017.11:g.31327673del	NM_001042492.3:c.5443del	NP_001035957.1:p.(Gln1815ArgfsTer48)	1	Likely Pathogenic<br><br>PVS1, PM2<br><br>(novel variant)	PlNf brachial<br><br>Fasi<br><br>Angiodysplasia
NC_000017.11:g.313	NM_001042492.3:c.7232_7233del	NP_001035957.1:p.(Arg2411AsnfsTer10)	1	**Pathogenic**<br><br>PVS1, PM2, PS4, PP5	PlNf intra-cranial

49162_313 49163del					Fasi
NC_000017.11:g.31206297C>T	NM_001042492.3: c.1318C>T	NP_001035957.1:p.(Arg440Ter)	2	**Pathogenic**<br><br>PVS1, PM2, PM1, PS4, PP5	PlNf gigantic femoral, malignance<br><br>PlNf neck
NC_000017.11:g.31261733C>T	NM_001042492.3: c.4600C>T	NP_001035957.1:p.(Arg1534Ter)	2	**Pathogenic**<br><br>PVS1, PM2, PS4, PP5	PlNf elbow & paravertebral (1 patient)<br><br>ADHD<br><br>c-l m<br><br>fasi
NC_000017.11:g.31338092C>T	NM_001042492.3: c.6772C>T	NP_001035957.1:p.(Arg2258Ter)	2	**Pathogenic**<br><br>PVS1, PS2, PM2, PP5	Symptomatic epilepsy<br><br>SpNf<br><br>Huge PlNf nose & choanae<br><br>Fasi
NC_000017.11:g.31334880G>A	NM_001042492.3: c.5855G>A	NP_001035957.1:p.(Trp1952Ter)	1	**Pathogenic**<br><br>PVS1, PM2, PS4, PP5	PlNf periorbital and temporal<br><br>SNf<br><br>fasi
NC_000017.11:g.31201471C>T	NM_001042492.3: c.1246C>T	NP_001035957.1:p.(Arg416Ter)	2	**Pathogenic**<br><br>PVS1, PM2, PS4, PP5	epilepsy<br><br>c-l m, fasi

NC_000017.11:g.31201471C>T	NM_001042492.3:c.1246C>T	NP_001035957.1:p.(Arg416Ter)	3	**Pathogenic**<br><br>PVS1, PM2, PS4, PP5	PlNf chest<br><br>ON & chiasma glioma<br><br>Fasi
NC_000017.11:g.31163231C>T	NM_001042492.3:c.334C>T	NP_001035957.1:p.(Gln112Ter)	1	**Pathogenic**<br><br>PVS1, PM2, PS4, PP5	PlNf paravertabral & paratracheal<br><br>Scoliosis<br><br>Lower paraparesis
NC_000017.11:g.31249042A>T	NM_001042492.3:c.4033A>T	NP_001035957.1:p.(Lys1345Ter)	1	Likely Pathogenic<br><br>PVS1, PM2<br><br>(novel variant)	PlNf sphenoid bone<br><br>ON glioma<br><br>femoral shortening
NC_000017.11:g.31338739C>A	NM_001042492.3:c.6855C>A	NP_001035957.1:p.(Tyr2285Ter)	2	**Pathogenic**<br><br>PVS1, PP1, PS2, PM2, PS3, PP5	PlNf occipital, neck<br><br>Diabetes<br><br>Myopia
NC_000017.11:g.31334966dup	NM_001042492.3:c.5941dup	NP_001035957.1:p.(Met1981AsnfsTer5)	1	Likely Pathogenic<br><br>PVS1, PM2<br><br>(novel variant)	PlNf face & cranial
NC_000017.11:g.31200499dupT	NM_001042492.3:c.966dup	NP_001035957.1:p.(Ala323CysfsTer7)	1	Pathogenic<br><br>PVS1, PM2, PP5<br><br>(novel variant)	PlNf thoracic region<br><br>fasi

NC_000017.11:g.31200553dup	NM_001042492.3:c.1020dup	NP_001035957.1:p.(Val341CysfsTer12)	1	**Pathogenic**<br><br>PVS1, PM2, PS4, PP5	PlNf<br><br>c-l m
NM_001042492.3:c.6383dup	NM_001042492.3:c.6383dup	NP_001035957.1:p.(Asn2128LysfsTer15)	1	Pathogenic<br><br>PVS1, PM2, PP5	PlNf periorbital and temporal
NC_000017.10:g.29662001dup	NM_001042492.3:c.5958dup	NP_001035957.1:p.(Gln1987ThrfsTer22)	1	Pathogenic<br><br>PVS1, PM2, PS4, PP5	PlNf mastoideus & near ear
NC_000017.10:g.29559168_29559169insGTGG	NM_001042492.3:c.3275_3276insGTGG	NP_001035957.1:p.(Val1093TrpfsTer14)	1	Likely Pathogenic<br><br>PVS1, PM2<br><br>(novel variant)	PlNf periorbital fasi

Table S2. Single nucleotide substitutions (missense variants) identified in NF1 patient cohort.

Genomic	Point variants	Protein	Quantity of repeats	Pathogenicity	Clinical features
NC_000017.11:g.31181755C>A	NM_001042492.3:c.700C>A	NP_001035957.<br><br>1:p.(Leu234Met)	3	Likely Pathogenic/VuS<br><br>(PM2, PP3, PM1, PP2, PP4)	PlNf pelvic & paravertebral (2 patients) with severe pain<br><br>1 patient diabetes mellitus

NC_000017.11:g.31201462T>C	NM_001042492.3: c.1237T>C	NP_001035957.<br>1:p.(Ser413Pro)	1	**Pathogenic**<br>PS4, PM2, PM1, PP3, PP2, PP5	PlNf
NC_000017.11:g.31214524A>G	NM_001042492.3: c.1466A>G	NP_001035957.<br>1:p.(Tyr489Cys)	2	**Pathogenic**<br>PS4, PM2, PM1, PP2, PS3, PP1, PP5	PlNf (1 patient)<br>c-l m
NC_000017.11:g.31221929G>A	NM_001042492.3: c.1721G>A	NP_001035957.<br>1:p.(Ser574Asn)	1	**Pathogenic**<br>PS2, PM2, PM5, PM1, PP2, PP5	PlNf extracranial above pr. Mastoideus
NC_000017.11:g.31229155T>C	NM_001042492.3: c.2540T>C	NP_001035957.<br>1:p.(Leu847Pro)	1	**Pathogenic**<br>PP1, PS2, PM2, PM5, PM1, PP3, PP2, PS3, PP5	ON glioma<br>Strabismus<br>Fasi
NC_000017.11:g.31258404A>G	NM_001042492.3: c.4234A>G	NP_001035957.<br>1:p.(Arg1412Gly)	1	**Pathogenic**<br>PS4, PM2, PM5, PM1, PP3, PP2, PS3, PP5	Symptomatic epilepsy<br>Mental retardation<br>Fasi<br>SNf
NC_000017.11:g.31259039A>C	NM_001042492.3: c.4340A>C	NP_001035957.<br>1:p.(Gln1447Pro)	1	**Pathogenic**<br>PS1, PP3, PM2, PM5, PM1, PS4, PP2, PP5	Scoliosis<br>Fasi<br>Epilepsy<br>SNf

NC_000017.11:g.31337404A>C	NM_001042492.3: c.6401A>C	NP_001035957.1: p.(Glu2134Ala)	2	VUS (novel variant)<br><br>PM2, PP3, PM1, PP2	PlNf occipital, neck & armpit area<br><br>PlNf neck

Table S3. Exon-intron splicing site variants in patients with *NF1*

Genomic	Donor-acceptor site	Quantity of repeats	Pathogenicity	Clinical features
NC_000017.11:g.31337881G>C	NM_001042492.3:c.6704+1G>C	1	**Pathogenic**<br><br>PVS1, PM2, PS4, PP5	PlNf elbow<br><br>fasi
NC_000017.11:g.31227217G>T	NM_001042492.3:c.2252-1G>T	1	Pathogenic<br><br>PVS1, PM2, PS4, PP5	PlNf lower jaw<br><br>Symptomatic epilepsy<br><br>fasi
NC_000017.11:g.31163378T>A	NM_001042492.3:c.479+2T>A	1	**Pathogenic**<br><br>PVS1, PM2, PS4, PP5	PlNf parotid and submandibular region<br><br>fasi
NC_000017.11:g.31337881del	NM_001042492.3:c.6704+1del	1	**Pathogenic**<br><br>PVS1, PM2, PS4, PP5	PlNf gluteal region<br><br>fasi
